# Understanding the Relevance of DNA Methylation Changes in Immune Differentiation and Disease

**DOI:** 10.3390/genes11010110

**Published:** 2020-01-18

**Authors:** Carlos de la Calle-Fabregat, Octavio Morante-Palacios, Esteban Ballestar

**Affiliations:** Epigenetics and Immune Disease Group, Josep Carreras Leukaemia Research Institute (IJC), 08916 Badalona, Barcelona, Spain; cdelacalle@carrerasresearch.org (C.d.l.C.-F.); omorante@carrerasresearch.org (O.M.-P.)

**Keywords:** epigenetics, DNA methylation, immune system, B cells, T cells, NK cells, innate lymphoid cells, monocytes, granulocytes, rheumatoid arthritis, systemic lupus erythematosus, cryopyrin-associated periodic fever syndrome, Familial Mediterranean Fever, primary immunodeficiencies, innate immune deficiencies

## Abstract

Immune cells are one of the most complex and diverse systems in the human organism. Such diversity implies an intricate network of different cell types and interactions that are dependently interconnected. The processes by which different cell types differentiate from progenitors, mature, and finally exert their function requires an orchestrated succession of molecular processes that determine cell phenotype and function. The acquisition of these phenotypes is highly dependent on the establishment of unique epigenetic profiles that confer identity and function on the various types of effector cells. These epigenetic mechanisms integrate microenvironmental cues into the genome to establish specific transcriptional programs. Epigenetic modifications bridge environment and genome regulation and play a role in human diseases by their ability to modulate physiological programs through external stimuli. DNA methylation is one of the most ubiquitous, stable, and widely studied epigenetic modifications. Recent technological advances have facilitated the generation of a vast amount of genome-wide DNA methylation data, providing profound insights into the roles of DNA methylation in health and disease. This review considers the relevance of DNA methylation to immune system cellular development and function, as well as the participation of DNA methylation defects in immune-mediated pathologies, illustrated by selected paradigmatic diseases.

## 1. Introduction: DNA Methylation Is a Cornerstone Regulator in Health and Disease

Cellular epigenomic landscapes are composed of a complex, interconnected, and extremely plastic network comprising DNA methylation, post-translational modification of histones and non-coding RNAs [1]. Increasing scientific effort to characterize transcriptional regulation at the molecular level has revealed even greater complexity through the discovery of higher-order layers of regulation, including three-dimensional chromosome organization, chromatin accessibility and RNA epigenetic modifications (epitranscriptomics) [2,3,4]. The development of high-throughput technologies has facilitated the generation of vast amounts of public epigenome-wide datasets that enable less biased and more integrative perspectives on how this regulation works in health and disease. Currently, state-of-the-art, single-cell sequencing technologies allow parallel multi-omics analyses [5,6] that are poised to uncover epigenetic heterogeneity at the level of resolution of the individual cell. 

DNA methylation, particularly cytosine methylation, is the best-studied epigenetic modification. It consists of the addition of a methyl group to the carbon 5 (5meC) of cytosine-followed-by-guanine dinucleotides (CG or CpG sites). It is characterized by its stability and heritability, which make it easy to analyze and interpret in several contexts. Initially, DNA methylation was mainly studied in the context of CpG-rich regions, known as CpG islands (CGI) [7], which are found in many vertebrate gene promoters. In the context of CGI, DNA methylation is generally associated with transcriptional repression and is pivotal in long-term gene silencing, X-chromosome inactivation, genomic imprinting and pre-mRNA alternative splicing [1,8]. However, more recent studies have highlighted the regulatory role of DNA methylation underlying dynamic biological processes [9]. These regulatory dynamics operate in gene bodies, enhancers, and partially-methylated domains (PMDs) [10,11,12]. Among different tissues and conditions, differentially methylated regions (DMRs) in regulatory sequences co-localize with transcription factor binding sites (TFBSs), which allows the driver regulators of these processes to be identified [13]. Furthermore, the DNA binding capacity of many transcription factors (TFs) can be directly or indirectly affected by the methylation of their binding sequences [14,15]. Moreover, a subset of TFs that possess the ability to rearrange the epigenome (so-called *pioneer* TFs) are able to interact with the epigenetic machinery, altering the DNA methylation status of their target regions [16,17]. The study of that bidirectional interplay enables us to describe genome-environment interactions at the molecular level in a variety of human biological contexts. 

The addition of methyl groups to DNA is mediated by DNA methyltransferases (DNMTs). DNMT1 is essential for the maintenance of DNA methylation following replication through cell division, while DNTM3A and DNMT3B are mainly responsible for *de novo* methylation. Passive DNA demethylation can occur after consecutive cell divisions. Active demethylation is mediated by members of the Ten-Eleven Translocation (TET) family, which includes TET1, TET2, and TET3. These oxygen-dependent enzymes catalyze the oxidation of 5meC to 5-hydroxymethylcytosine (5hmc), 5-formylcytosine (5fC), and 5-carboxylcytosine (5caC). 5fc- and 5caC-modified positions are bound and excised by the thymidine-DNA glycosylase (TDG) enzyme, yielding an abasic site that is replaced with an unmethylated cytosine by base-excision repair (BER). Recent studies have described certain stable genome-wide distributions of 5meC demethylation intermediates, although the role of these modifications as autonomous epigenetic marks is still a matter of debate [18]. 

Given the complexity of epigenomic regulation, it is not surprising that there are opposing opinions about the capacity of 5meC on its own to regulate gene expression. A bioinformatic reanalysis of data from a recent study showed that forced genome-wide hypermethylation can, on its own, repress gene expression and rewire chromatin to an inactive state [19,20]. 

The relevance of 5meC in genomic regulation in all the aforementioned contexts shows it to be a pivotal regulator of human cell biology. Thus, the alteration of 5meC homeostasis is usually synonymous with pathology, and has been linked to numerous diseases, like cancer, developmental disorders, and immune diseases [21,22,23]. 

Many studies have shown that immune cell differentiation, identity, and function require fine-tuning epigenetic regulation [24]. The study of patients with defects in these processes allows the identification and characterization of epigenetic mechanisms and novel environment-genome interactions. As a consequence, the importance of DNA methylation as a biomarker has become more widely appreciated, especially for complex diseases that cannot be entirely accounted for by genetic associations [25]. In this review, we outline the latest advances in our understanding of the role of DNA methylation in immune system function. We also examine a curated list of immune diseases, grouped by type, and their associated DNA methylation defects, with the aim of providing a perspective on the role of these alterations as pathogenic contributors or as potential clinical biomarkers. 

## 2. DNA Methylation in the Immune System

The broad functional diversity of cell types is highly dependent on various epigenetic mechanisms that regulate and modulate gene expression. These mechanisms are particularly relevant in the hematopoietic system due to the great complexity and abundance of its many cell types and subtypes. In hematopoiesis, immune cells are produced hierarchically from the same cell type: hematopoietic stem cells (HSCs), which are the only cell type with the capacity for self-renewal in the absence of differentiation. HSCs give rise to common lymphoid and myeloid progenitors that are the precursors, respectively, of all lymphoid (B, T, and NK cells) and myeloid cells (monocytes, neutrophils, eosinophils, basophils, and mast cells). 

DNA methylation regulation is essential for the differentiation of HSCs. The first whole DNA methylome maps indicated that differentiation from HSCs towards the lymphoid lineage increases levels of DNA methylation, in contrast to the decrease observed during the differentiation towards the myeloid lineage [26]. It has been shown that the loss of *Dnmt3a* expression in mouse HSCs impairs their differentiation and blocks the silencing of HSC multipotency genes. The concomitant loss of DNMT3B synergizes with that phenotype [27,28]. Moreover, loss-of-function mutations of TET enzymes in humans are associated with several myeloid malignancies, and the silencing of *Tet2* in mice also produces an increase in the hematopoietic stem cell compartment and myeloproliferation [29]. A recent study described how the loss of TET2 not only impairs demethylation of CpG sites, but also reduces the activity of TFs in enhancers in these cells [30].

**Figure 1:**
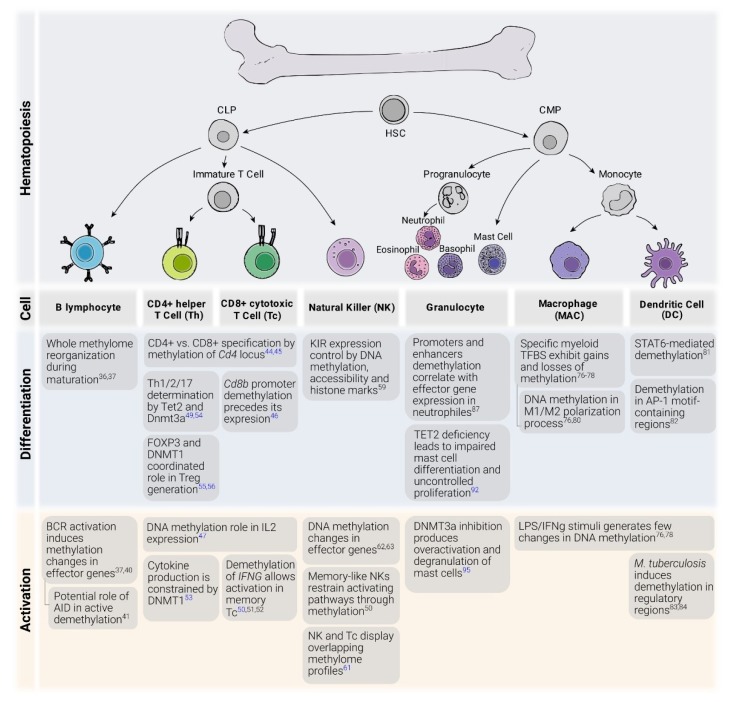
DNA methylation regulation processes in immune cell function, differentiation and activation. HSC: hematopoietic stem cell, CLP: common lymphoid progenitor, CMP: common myeloid progenitor, Th: CD4+ T helper cell, Tc: CD8+ cytotoxic T cell, NK: natural killer cell, MAC: macrophage, DC: dendritic cell. BCR: B cell receptor, KIR: Killer cell immunoglobulin-like receptor, TFBS: Transcription factor binding site, LPS: Lipopolysaccharide. In blue, references of studies in mice. In black, references of studies in human.

In the following sections, we describe the relevance of DNA methylation to the biological role of various immune cell types (a summary of major DNA methylation related events is shown in Figure 1).

### 2.1. B Cells

B cells, which are responsible for antibody-mediated immunity, experience a complex series of differentiation, maturation, and activation stages that represent a paradigm for studying epigenetic regulation dynamics. Many studies have described the variety of epigenetic patterns underlying these dynamics in different contexts [31,32,33,34,35], revealing vast amounts of reconfiguration, which is more common in B cells than among other types of immune cells [36]. 

From early differentiation in the bone marrow to their maturation in the spleen, human B cells undergo extensive changes in their DNA methylome, in up to one-third of all their CpGs. During development, moderate demethylation takes place in enhancer regions containing binding sequences of B cell lineage TFs. In this case, DNA methylation changes are infrequently correlated with gene expression, and are, thus, more likely to be regulatory and lineage-determining. After activation by B cell receptor (BCR) stimulation, large-scale demethylation at late-replicating domains and hypermethylation at polycomb-repressed regions occurs, arguably as a consequence of the increase in the proliferation rate and an aging-associated epigenetic drift, respectively [37,38,39]. Similarly, for some types of leukemia, malignant cells display a methylome that reflects the normal developmental stage from which they are derived. These tightly defined and stable epigenomic profiles allow patients to be stratified and their outcomes predicted [40].

After BCR activation by specific antigens, somatic hypermutation (SHM) and class-switch recombination (CSR) events occur. These are required for affinity maturation and antibody diversification within the germinal center (GC) in the spleen. Both processes are orchestrated by the activity of activation-induced cytidine deaminase (AID) enzyme, which catalyzes the deamination from C to U, which after restoration through BER leads to changes in the sequence. In addition to these mechanisms, AID, which is sensitive to methylated C, has been proposed to play a role in active demethylation by deamination of 5meC and 5hmeC [41], although the evidence is contradictory, meaning that further studies are needed to settle the matter [42]. However, AID deficiency in humans causes overstimulation of B cell proliferation, leading to aberrant DNA methylation and gene expression profiles in naïve and memory B cells [43].

### 2.2. T Cells

The adaptive immune system requires the rigorous control of the epigenome for both cell fate specification and activation. In mice, it has been shown that specifying the T cell subtype as helper (CD4+) or cytotoxic (CD8+) from thymocytes involves DNA methylation changes. Specifically, during lineage choice, dynamic DNA methylation of the *Cd4* locus is essential for stable CD4 expression in CD4+ cells and its repression in CD8+ cells, which is orchestrated alongside lineage-specifying TFs in a heritable fashion [44,45]. Concomitantly, CD8 co-receptor gene *Cd8b* undergoes promoter hypomethylation, showing an inverse correlation with expression in thymocytes and lymph node CD8+ T cells [46].

Following T cell receptor (TCR) stimulation, interleukin-2 (IL2) expression is required for T cell activation and proliferation. Active demethylation of the *Il2* locus is required for stable IL2 expression in activated mouse T cells [47]. Upon activation, Th cells can polarize into Th1, Th2, and Th17 lineages, with different immune functions and expression of cytokines [48]. The transcriptional regulation of these specific cytokine genes is also regulated by active demethylation by TET2, which is recruited by key TFs, like T-bet, RORγt, and STAT4. The expression of cytokines IFNγ, IL4, and IL17, hallmarks of Th1, Th2, and Th17, respectively, are impaired in TET2^−/−^ mice, which appears to be concomitant with a decrease in 5hmeC in their loci [49]. Active demethylation of the *Ifng* locus is also observed in the transition from naïve to memory CD8+ T cells, which allows increased IFNγ secretion in effector memory cells [50]. These changes have also been observed in human memory cell subsets, reinforcing the notion of a specific methylome profile as a priming mechanism for the activation of resting memory cells. Similar to mice, methylation patterns are stably located at effector loci and remain poised for a secondary activation of central memory CD8+ T cells [51,52]. 

DNMTs also regulate T cell cytokine expression. DNMT1 is necessary to restrain cytokine expression in proliferating activated Th cells [53]. It has also been proposed that DNMT3a, which is highly overexpressed after TCR stimulation, provides a fine-tuning mechanism for Th1/Th2 stable selection by hypermethylating specific subtype cytokine loci in mice [54]. 

Regulatory T cells (*Tregs*), identified by the expression of TF FOXP3, are generated in the thymus as a distinct lineage, with immunosuppressive functions that limit spurious autoimmunity and inflammatory excess. DNMT1 cooperates with FOXP3 and other TFs to differentiate Tregs. In fact, conditional knockout of DNMT1 in mouse T cells impairs global DNA methylation maintenance, altering the transcriptional program of Tregs, leading to lethal autoimmunity [55,56]. 

### 2.3. Innate Lymphoid Cells

Innate lymphoid cells (ILCs) are a heterogeneous group of generally tissue-resident cells that arise from common lymphoid progenitors (CLPs) and comprise natural killer (NK) cells as well as non-cytotoxic ILCs, classified in three groups (ILC1, ILC2 and ILC3) depending on their location, surface protein expression, and the involvement of distinct TFs during their differentiation. ILCs have recently been reported to have a role in a variety of contexts, such as gut immunity, allergy, autoimmunity, and metabolism homeostasis, essentially through cytokine production [57,58].

NK cells are the best studied type of ILC. In a similar way to adaptive immunity, their activation is fast and their cytotoxic activity involves tumoral and virus-infected cells that have impaired surface expression of major histocompatibility complex protein I (MHC-I). These MHC-I-directed cytotoxicity is mediated through NK killer-cell Immunoglobulin-like receptor (KIR) family. In hematopoietic progenitors, KIR genes loci retain a highly methylated status, alongside repressive histone modifications and reduced chromatin accessibility. In KIR-expressing cells, the specificity of each KIR gene expression is determined by hypermethylation and repressive histone marks of the other KIR loci [59].

In some cases, virus infection can lead to the expansion of memory-like NK cells, which remain at rest by the inhibition of NK-activating pathways concomitant with hypermethylation of their component loci [60]. Memory-like NK cells have been shown to display similar DNA methylation profiles to that of memory CD8+ T cells, overlapping with lymphocyte-activating pathways in mice [61]. In line with these results, methylome profiling of human in vitro-activated NK cells has revealed demethylation in promoters of cytokines including IFNγ, IL13, and IL5, similar to what occurs after T cell activation [62,63].

There is currently no available genome-wide DNA methylation data from non-cytotoxic ILC subtypes, although the characterization at the transcriptional and histone modification levels has shed light on the regulatory mechanisms in ILCs [64,65,66], pinpointing the need to obtain methylomes of ILCs to better understand their biology.

### 2.4. Monocytes and Macrophages

Monocytes and macrophages, the main cell types representing the so called mononuclear phagocyte system, have complementary roles in immunological challenges. Monocytes, which make up 5–10% of leukocytes in peripheral human blood, differentiate from granulocyte-monocyte progenitors in the bone marrow. They can be classified into three main groups based on the expression of surface markers CD14 and CD16: classical monocytes (CD14++CD16−), intermediate monocytes (CD14+CD16+) and non-classic monocytes (CD14−CD16++). There are several lines of evidence to suggest these groups are not independent but, instead, different stages of differentiation of the same population [67,68].

Classical monocytes are the most abundant type (~85%). Even though most methylation studies focus on CD14+ monocytes, growing evidence of the relationship of non-classic and intermediate monocytes with inflammatory pathologies suggests that the study of methylation differences between these populations, in physiological and pathological contexts, is potentially revealing. For instance, raised frequencies of non-classic and intermediate monocytes have been described in a variety of inflammatory diseases, such as rheumatoid arthritis, sepsis, and systemic lupus erythematosus [69,70,71].

Although studies in mice suggest that most tissue macrophage populations are established before birth [72], mouse and human monocytes can migrate into tissues and differentiate into one of several cell types, such as mo-DCs (monocyte-derived dendritic cells) and mo-MACs (monocyte-derived macrophages), depending on the external stimuli, in chronic and acute inflammatory conditions, and in the steady-state [68,73].

In vitro models for differentiating macrophages from monocytes have been critical to progress in the field. TET2 is strongly expressed in these cells and many studies have shown the relevance of this enzyme in monocyte differentiation to macrophages. There are two main differentiation models of human monocytes to macrophages. The first involves treatment with the ubiquitous cytokine M-CSF, and is considered to produce ‘steady-state’ macrophages; the second involves GM-CSF, and the resulting macrophages are supposed to emulate those present in an inflammatory context [74]. Moreover, differentiation with GM-CSF and IL-4 is commonly used to produce dendritic cells in vitro [75].

M-CSF stimulation of monocytes results in gains and losses of DNA methylation in the resulting macrophages, but different studies disagree about the relative importance of the two processes. One showed that there are more than three times as many hypermethylated as hypomethylated CpGs [76]. However, most studies indicate that hypomethylation predominates over hypermethylation [77,78]. 

After differentiation, M-CSF macrophages can be polarized to an inflammatory or ‘M1’ state (LPS) or an anti-inflammatory ‘M2’ phenotype (IL-4) [79]. Methylation changes with this second insult have been found to be negligible in more than one occasion [76,80], suggesting that after the monocytes become committed to a differentiated stage, the methylation state is relatively stable, and the changes in the transcriptome are probably produced through other regulatory mechanisms.

On the other hand, GM-CSF produces fundamentally TET2-dependent hypomethylation, mostly in intergenic regions and gene bodies [81]. In comparison with GM-CSF + IL4, the activation of JAK3-STAT6 downstream of IL-4 triggers the DNA methylation changes following STAT6 binding. Further results pinpoint the presence of AP-1 TF binding motifs within the hypomethylated regions in mo-DC differentiation [82]. As previously described in M-CSF macrophages, the subsequent activation of GM-CSF mo-MACs and mo-DCs with LPS produces very few changes in DNA methylation [81]. 

Conversely, a recent study found significant DNA methylation changes after the activation of immature mo-DC through infection with live bacteria (*Mycobacterium tuberculosis*, MTB) [83]. The use of a live and virulent strain of MTB instead of LPS could explain this discrepancy with the previously mentioned study, because the signaling could operate through different pathways. Moreover, the incubation time (72 h) is longer than in previous studies, and the platform used was a CpG custom panel of 33,059 regions with H3K4me1 signal, which was associated in a previous study with the methylation changes in infection [84]. However, the authors reported that DNA methylation changes due to infection are a consequence rather than a cause of the transcriptional changes, suggesting that DNA demethylation is mediated by prior TF binding. As the majority of differentially methylated CpGs gradually and irreversibly reduce the methylation level, at least during the first 72 h, the authors proposed that they could be relevant to the response to future infections, as a mechanism of innate immune memory [85].

Another monocyte-related differentiation model showed that the TF PU.1 can interact with DNMT3b and TET2 to alter DNA methylation and expression [86]. DNA became hypomethylated in genomic regions associated with osteoclastogenesis, whereas hypermethylation could silence genes involved in other differentiation processes.

### 2.5. Granulocytes

Granulocytes are cells from the innate immune system, characterized by the presence of granules in their cytoplasm. They are also known as polymorphonuclear leukocytes or polymorphonuclear neutrophils because of the varying shapes of their nuclei. Granulocytes include neutrophils, eosinophils, basophils, and mast cells, and they are involved in phagocytosis of bacteria, killing of pluricellular parasites, and histamine release during inflammation in blood and tissues, respectively. The majority of existing research on the epigenetics of granulocytes has centered on neutrophils and mast cells, so evidence about the relationship of DNA methylation and eosinophil/basophil biology is scarce. 

Research into DNA methylation of human granulopoiesis has shown that successive waves of hypermethylation and hypomethylation occur in early and late developmental stages, respectively. Transcriptional activation of effector neutrophil genes is preceded by demethylation of their promoters, although the majority of the methylation losses take place in enhancer regions [87]. Several authors have emphasized that neutrophils display the highest number of hypomethylated regions of all blood cell types, in keeping with their fully differentiated and effector phenotype [88,89,90]. 

Mast cells are key effectors of allergy and immunity to parasites. They are strictly tissue-resident cells, which makes their isolation difficult [91]. Evidence arising from two studies of the same group suggests an overarching role for methylation machinery in mast cell biology in mice, involving both TET2 and DNMT3a. Tet2-deficient mice have impaired differentiation and uncontrolled proliferation of mast cells [92]. Interestingly, TET2 loss-of-function mutations are present in a considerable percentage of patients with mastocytosis [93,94]. On the other hand, neither DNMT3a-deficient nor DNMT inhibitor-treated mice exhibited altered proliferation but led to overactivation and degranulation of mast cells in vitro and in vivo [95].

## 3. DNA Methylation in Immune-Mediated Diseases

Immune system responses can be divided into two main groups: innate immunity and adaptive immunity. Innate immune responses provide a rapid reaction following the recognition of generic patterns of pathogens, while adaptive immune responses can recognize any type of molecular structure, in a slow manner, and promote long-term immune memory. Although there is continuous crosstalk between the different cell types of the immune system, T and B cells are considered the main effectors of adaptive immunity, whereas monocytes, granulocytes, and ILCs drive innate immune responses.

Since the establishment of correct DNA methylation patterns is linked to the proper differentiation and function of immune cells, it can be assumed that dysregulation of the DNA methylation machinery in these cells can produce immune system disorders, and that immune dysfunction may be reflected in the generation of abnormal patterns of methylation.

Monogenic diseases constitute excellent model systems for interrogating specific mechanisms associated with a given gene. However, many immune diseases are associated with the combination of many genetic susceptibility variants conferring susceptibility, and it is usually difficult to isolate the specific contribution of each of these genes. This is the case for the majority of autoimmune diseases and for some non-monogenic immunodeficiencies [96]. Moreover, the existence of high rates of discordance in monozygotic (MZ) twins for these diseases, reinforces the effect of the environment on disease etiology [97]. 

**Figure 2:**
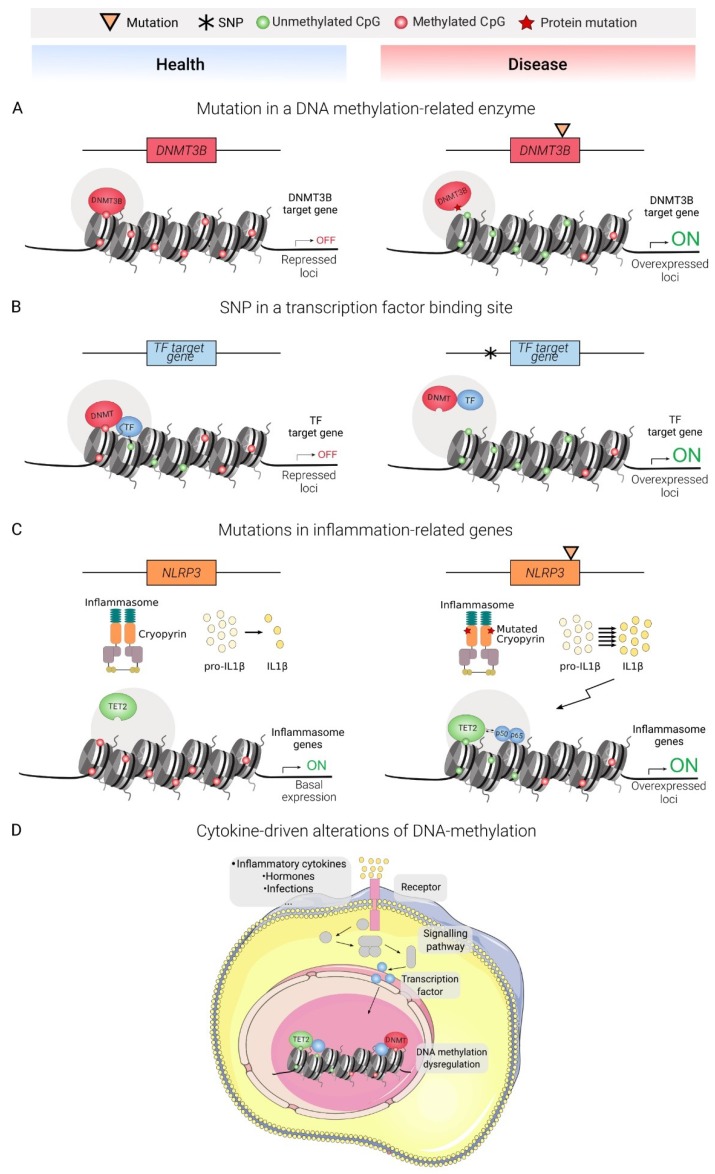
Proposed theoretical models of association between genetic and epigenetic determinants of immune diseases. (**A**) Mutations in genes encoding for components of the DNA methylation machinery, (**B**) polymorphisms in TFBS altering TF binding capacity, and (**C**) mutations in inflammation-related proteins with impact on DNA methylation. (**D**) External inflammatory stimuli with impact in DNA methylation.

**Figure 3:**
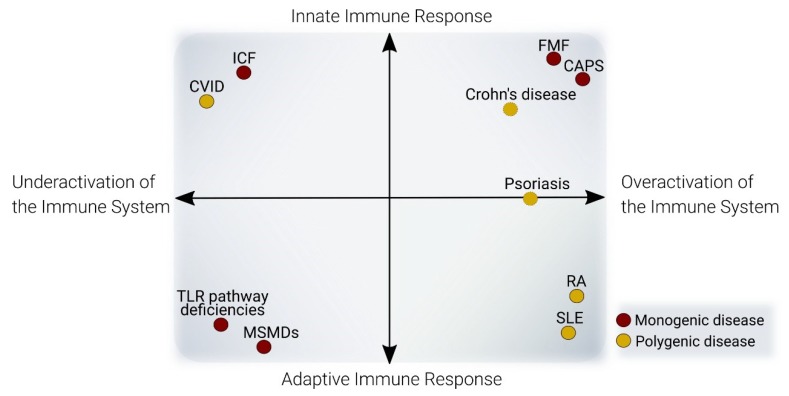
Classification recommendation of immune system diseases: This graph depicts a proposed methodology for the classification of immune diseases, accounting for type of response affected (innate/adaptive) and effect of the dysfunction in activity (underactivation/overactivation). Each quadrant discriminates cases of diseases with diverse implications regarding the two axes. Dot color indicates the number of genes affected in each disease: red for monogenic diseases, yellow for polygenic diseases. Dashed points: examples of diseases not further detailed in the text. CVID: Common variable immunodeficiency, TLR: Toll-like receptor; ICF: Immunodeficiency-centromeric instability-facial anomalies; MSMD: Mendelian susceptibilities to mycobacterial disease; FMF: Familial Mediterranean Fever; CAPS: Cryopyrin-associated periodic syndromes; SLE: Systemic Lupus Erythematosus; RA: Rheumatoid arthritis.

Several lines of evidence suggest that genetic and epigenetic alterations can be functionally connected in diseases (Figure 2). For example, an increasing amount of epigenomic data concerning complex diseases has allowed the generation of genomic-epigenomic integrative approaches for the discovery of novel disease associated-loci, termed *meQTL* (methylation-quantitative trait loci) [98,99]. The following section presents an updated review of DNA methylation alterations of representative immune diseases, comprising overactivation and deficient activity of the adaptive and innate immune systems (Figure 3 and Table 1).

### 3.1. DNA Methylation Defects in Conditions with Overactivation of the Immune System

#### 3.1.1. Autoimmune Diseases

Autoimmune diseases are the most common disorders of the immune system. Patients are generally characterized by overactivation of adaptive immune responses, although, in some diseases in this group, cells from both innate immune systems are also affected. They are mostly polygenic, and familial heritability accounts for only a minority of cases. Furthermore, they can be associated with environmental disease risk factors. For all these reasons, there has been great scientific interest in studying the epigenetic alterations of patients, whereas classic genome-wide association studies (GWAS) have failed to provide a final explanation of the complete basis of diseases. In this section, we provide an updated review of two high-incidence autoimmune rheumatic diseases, rheumatoid arthritis (RA), and systemic lupus erythematosus (SLE). These are the two best studied autoimmune diseases from an epigenetics standpoint.

##### Rheumatoid Arthritis

RA is a chronic autoimmune disease that primarily affects joint synovium, although patients can also manifest systemic symptoms. It affects up to 1% of the population, with a 3:1 female:male sex bias. Familial heritability of the disease is relatively low and accounts for only 20–50% of cases, at most, depending on the disease subtype [123]. Environmental factors, such as cigarette smoke, are known to be disease risk factors, although most of them are yet to be identified [124]. 

A considerable number of existing studies have found epigenetic alterations in peripheral blood cell populations, reinforcing the idea of RA being a disease with local and systemic affectation. Genome-wide DNA methylation analysis of peripheral blood lymphocytes established an association between methylation, genotype, and disease status. Identified disease-associated differentially methylated positions (DMPs) in the MHC locus were proposed as disease mediators of genetic predisposition [98]. Methylation levels of specific loci have also been linked to the response to treatment with disease-modifying antirheumatic drugs (DMARDs) in T cells [101], and etanercept (a TNF inhibitor) in whole blood [100]. A recent study demonstrated that DNA methylation in *CYP2E1* and *DUSP22* genic regions is correlated with disease activity and erosivity, respectively, in several cell types [102]. The results in that study might be limited to a few regions due to a lack of statistical power arising from the small number of high-activity patients in the analyzed cohort. However, in a subsequent study of a disease activity-balanced cohort, we found strong associations between disease activity and methylation in peripheral blood monocytes, arising from the influence of proinflammatory cytokines in blood serum. Methylation levels in those regions were largely reversible during patient evolution over time. These associations made it possible to generate a predictive mathematical model to estimate patient activities from DNA methylation values [103]. 

Other studies have analyzed synovial populations. However, genome-wide DNA methylation profiling has only been conducted in fibroblast-like synoviocytes (FLSs). Comparison of FLSs between RA and OA (osteoarthritis, or non-rheumatic arthritis) revealed differential methylation among these conditions [104]. However, another study determined that FLS methylome profiles of RA were more similar to those of healthy controls than of OA [105]. Those results call into question the use as bona fide controls of OA FLS, which also display an altered phenotype. Finally, an extensive multi-omics characterization of synovial fibroblasts has been recently generated, revealing a complex epigenetic alteration of the disease-related pathways [106]. Single-cell RNA-seq and mass cytometry characterization of synovial fluid cells have recently revealed an intricate scenario with multiple RA-associated cell states, identified for the first time [107]. These data make it possible to identify new cellular drivers of disease and to carry out a detailed dissection of new etiopathological molecular pathways.

##### Systemic Lupus Erythematosus

SLE is a complex autoimmune disease with a poorly characterized etiology. It affects multiple organs and the most common symptoms include body pain, fever, and neuropsychiatric syndromes. As with other complex diseases, despite considerable scientific effort, genetic association studies have failed to determine disease heritability for most cases. As in other autoimmune diseases, environmental exposure to ultraviolet light, smoke, and emotional stress have been associated with a disease risk. They are probably mediated through epigenetic mechanisms, which act as a bridge between the environment and phenotype, and may help to explain the lack of heritability [125]. SLE is one of the diseases most thoroughly studied from an epigenetic point of view. However, it is worth noting that genetic variance, cell type composition, and the effect of covariates, such as treatments, which are all known to induce epigenetic alterations, may limit the interpretation of epigenome-wide association studies (EWAS), as well as their conclusions about causality [16,126]. This has provoked controversy in the scientific community and may relegate EWAS data to use in biomarker discovery rather than for elucidating biological mechanisms.

The first high-throughput analysis of DNA methylation in SLE made use of MZ discordant-twin whole-blood samples, identifying alterations of several genes of the IFN type I regulon for the first time [127]. These data have been confirmed by a recent study in whole blood of a much larger cohort of SLE patients, which described a large list of novel DMPs, exhibiting the most significant changes in the IFN type I target genes. Further, their data have been integrated with SLE-associated SNPs, revealing new methylation quantitative trait loci (meQTLs), most of which are located in the HLA type II locus [108]. Another study revealed strong differences underlying disease activity subtypes and ethnicity, which have been shown to affect SLE severity notably [109].

Despite an observed general effect in whole-blood-cell analyses, specific cell types are known to predominantly mediate pathology in SLE. The expansion of autoreactive CD4+ T cells leading to overactivation of B cell humoral responses is a hallmark of SLE, leading to CD4+ T cells becoming the best-studied cell type. Consistent with the aforementioned results, genome-wide analysis of DNA methylation in CD4+ T cells revealed hypomethylation of IFN type I of SLE patients, concomitant with a gain of 5hmC in those regions, followed by transcriptional activation [110,111]. Data from several authors indicated that this hypomethylation is also present in other cell types like B cells and monocytes, in a stable fashion, regardless of the clinical stage of the patient. These data suggest that quiescent patients could be more sensitive to IFN type I signaling in future active stages [112]. Further, DNA hypermethylation in SLE has been found specifically in B cells [113]. 

IFNα from the IFN type 1 family has been shown to induce DNA methylation changes in monocytes in vitro [103]. Taken together, these data imply that the epigenetic alterations of the IFN type I genes in several cell types are likely to be a consequence of an aberrantly active IFN-I pathway in SLE. 

#### 3.1.2. Autoinflammatory Diseases

Just as autoimmune diseases involve activation of the adaptive immune system, autoinflammatory diseases entail excessive activation primarily of the innate immune system. They are characterized by recurrent inflammation without an external trigger or the presence of high levels of autoantibodies and self-reactive T cells. Although most autoinflammatory diseases are considered monogenic diseases, such as Crohn’s and Behçet’s disease, are genetically complex. However, increasing evidence shows that even autoinflammatory monogenic diseases are influenced by the genetic background together with epigenetic and environmental factors that can determine the development of the disease. Even though we classified diseases related to overactivation of the immune system into autoimmune and autoinflammatory diseases, the actual context is more complex and implies a spectrum of diseases with features of both types. In the next section, we review DNA methylation in two paradigmatic autoinflammatory diseases. 

##### Cryopyrin-Associated Periodic Fever Syndrome

Cryopyrin-associated periodic fever syndrome (CAPS) is a group of autoinflammatory autosomal-dominant diseases characterized by gain-of-function mutations in the *NLRP3* gene, which produces the protein cryopyrin, a critical component of inflammasomes, which play a crucial role in processing precursors of IL1B, IL18, and IL33 to produce their mature forms [114,128]. Specifically, cryopyrin acts as a DAMP (damage-associated molecular pattern) sensor, and is able to initiate the assembly of the inflammasome [129]. These mutations produce overactivation of inflammasomes and, consequently, overproduction of IL1B.

Patients with CAPS suffer recurrent episodes of fever and IL1B-mediated systemic inflammation involving skeleton, joints, eyes, muscles, skin, etc. Three clinical manifestations are found in the CAPS family: familial cold autoinflammatory syndrome (FCAS), Muckle–Wells syndrome (MWS) and chronic, infantile, neurological, cutaneous and articular syndrome (CINCA) (also known as neonatal-onset multisystem inflammatory disease (NOMID)), in order of increasing severity [130].

Even though most CAPS mutations are located in exon 3, which contains the NACHT domain, which is important in cryopyrin oligomerization [130], some mutations have also been described in exons 4, 6, and 1. FCAS and MWS patients usually show familial inheritance patterns, whereas CINCA syndrome mutations usually occur de novo.

Although CAPS genetic bases are well known, and some mutations are consistently associated with a mild or severe phenotype, there are other mutations with very variable severities [131], suggesting that the genetic and epigenetic contexts could play a crucial role in the final outcome of the disease. 

In this regard, monocytes from CAPS patients are known to feature enhanced demethylation of inflammasome-related genes, concomitantly with an increase in gene expression [132]. Moreover, treating patients with anti-IL1 drugs produces a reversion in this abnormal methylation profile. Untreated CAPS patients with different mutations showed a similar methylation profile. As some patients fail to be diagnosed based on genetic criteria, DNA methylation could be used as an additional biomarker.

Furthermore, CINCA patients present differences of expression in DNA methylation-related genes such as *DNMT3L* and *TET2* between lesional and non-lesional skin [115].

##### Familial Mediterranean Fever

Familial Mediterranean Fever (FMF) is the best-known monogenic autoinflammatory disease and its symptomatology is characterized by recurrent fevers and chronic inflammation in joints, skin, and peritoneum [133].

The disease is associated with mutations in the *MEFV* gene, which codify the pyrin protein CITA. Some studies have suggested an anti-inflammatory role for pyrin [134], but it has since been hypothesized that a pyrin-driven inflammasome exists [135,136,137]. Additionally, a study in a murine model suggests a gain-of-function model of pyrin pathogenesis rather than one of loss-of-function [138]. 

Although FMF is usually considered an autosomal recessive disease [139], around 30% of patients present only one *MEFV* mutation [140,141,142], which means that environmental, genetic, and epigenetic factors could be important in the development of the disease.

The *MEFV* exon 2 was found to be marginally more methylated in peripheral leukocytes from FMF patients than in healthy controls, its expression level being negatively correlated with methylation [143]. These results have been validated in an in vitro model, which showed aberrant methylation in exon 2, which produces an increase in an aberrant spliced form of pyrin that also occurs at higher levels in FMF patients than in healthy donors [116,117]. Since this analysis of peripheral blood mononuclear cells (PBMC) shows differences in methylation, the results should be interpreted with caution. Patients could exhibit alterations in cell proportions. Moreover, DNA methylation changes could take place in a single cell type, and the analysis of mixed populations could dilute the effects occurring in a specific cell type.

For instance, neutrophils from FMF patients showed a lower level of *MEFV* gene expression compared with healthy donors, although there were no methylation differences [144]. Therefore, although DNA methylation could have a function in FMF pathology by altering the expression of pyrin, more precise studies are needed to determine its exact role.

### 3.2. DNA Methylation Defects in Conditions with a Deficient Immune System

#### 3.2.1. Adaptive Immune Deficiencies

Immunodeficiencies are characterized by an impairment of adaptive immune responses. They are classified with respect to the affected response component, and can be intrinsic (primary) or acquired (secondary), and monogenic or polygenic. Monogenic primary immunodeficiencies (PIDs) are very rare diseases that have mainly been used as models for studying immune system function. In this section, we review two examples of immunodeficiencies known to display alterations in DNA methylation: Immunodeficiency-centromeric instability-facial anomalies (ICF) syndrome, a monogenic PID characterized by mutations of the *DNMT3B* gene, and common-variable immunodeficiency (CVID), a complex disease with no common causative gene.

##### Immunodeficiency, Centromeric Region Instability, and Facial Anomalies Syndrome 

ICF is a paradigmatic example of a monogenic disease directly affecting a DNA methylation enzyme [22]. It is a very rare autosomal recessive condition, having been described in only around 60 patients worldwide [145]. It is characterized by hypoglobulinemia, despite the presence of B and T lymphocytes, facial anomalies, and centromere instability [118]. ICF patients also exhibit a loss in DNA methylation, especially in satellite repeats in pericentric regions [146].

Since the most consistent symptom of the ICF syndrome is hypoglobulinemia, characterized in particular by low serum levels of IgG and IgA, but also of IgM in most patients [118], B cells may be affected mainly in terms of their maturation or activation, rather than class switching [147]. An increase in T-cell apoptosis and a reduction in CD4+ cells with age have also been described in ICF syndrome [148,149].

Around 50% of ICF patients have a biallelic mutation in the DNMT3B gene. These mutations are heterogeneous among patients, but usually overlap with the catalytic domain of the protein [147]. ICF-specific rearrangements in mitogen-treated lymphocytes of patients are similar to those produced in pro-B lymphoblastoid cells treated with DNA methylation inhibitors, such as 5-aza-cytidine [150], and the catalytic assay of six mutations of *DNMT3B* in ICF patients showed a dramatic reduction in methylation activity [151], providing evidence that the main role of DNA methylation dysregulation in the pathological mechanisms of the disease. However, as the knock-out of *Dnmt3b* in mice produces prenatal death [152], ICF syndrome patients probably conserve some residual activity of DNMT3b, concordantly with in vitro assays and mouse models of ICF mutations [151,153].

DNA methylation and gene expression have been studied in ICF syndrome patients, using immortalized lymphoblastoid cell lines (LCLs). Whole-genome bisulfite sequencing revealed a 42% decrease in the global DNA methylation level, the reductions being especially common in inactive heterochromatic regions, transposons, and satellite repeats [154]. Methylation alterations were also detected in promoters of genes related to immune function and B-cell maturation, as previously described [155].

Some genes upregulated in ICF syndrome patients, such as *PTPN13*, *LHX2*, *PRRX1*, and *HHEX*, have lower levels of DNA methylation, are enriched in transcriptionally active histone marks and less binding of DNMT3b, and SUZ12 (a component of PRC2 polycomb repression complex), suggesting a connection between DNMT3b and polycomb-mediated repression [155]. Another study found a decrease in DNA methylation in LCLs of ICF syndrome patients near genes related to germline function (*BOLL*, *SYCP2*, *LDHAL6A*, and *NCRNA00221*) and an increase in mRNA levels [145], concordantly with a previous report of germline gene hypomethylation in ICF syndrome patients [119].

ICF syndrome patients can be classified based on the impaired gene, in addition to *DNMT3B* (ICF1): *ZBTB24* (zinc-finger and BTB domain-containing 24, ICF2), *CDCA7* (cell division cycle-associated 7, ICF3), and *HELLS* (helicase, lymphoid-specific, ICF4) [120,156,157]. Although the relationships of ZBTB24, CDCA7, and HELLS with DNA methylation are not completely understood, the similarities between ICF syndrome types indicate a possible role for the different genes in the same molecular pathway.

For instance, ZBTB24 has been described as a TF with target genes that are partially coincide with those of DNMT3B. Both proteins interact and the depletion of ZBTB24 reduces DNMT3b occupancy in common target genes, which suggests that ZBTB24 has a role in recruiting DNMT3b [158]. Some ICF2 missense mutations were found to alter zinc fingers relevant to the specific DNA binding of ZBTB24 [159].

Furthermore, HELLS is an SNF2 ATPase protein required for *de novo* demethylation in mice [160]. HELLS is able to interact with DNMT3b and its knock-out produces genome-wide demethylation [161,162]. CDCA7, which is also a target gene up-regulated by ZBTB24 [163], is required for HELLS to bind their targets in the genome and initiates their nucleosome remodeling activity [164]. These data ultimately link all the ICF-related genes and support the hypothesis of a common pathway.

Conversely, different methylation profiles have been reported between ICF1 and ICF2/3/4 patients, respectively [165]. Even though pericentromeric repeats and PCDH (protocadherin) genes are equally hypomethylated in all ICF syndrome patients, *ZBTB24*, *CDCA7*, and *HELLS* mutations mainly affect heterochromatic CpG-poor regions (open seas), some of which are associated with genes related to neuronal development, which is consistent with the more pronounced mental retardation in these subtypes of patients [166]. Although this does not necessarily rule out the existence of a common route comprising DNMT3B, ZBTB24, CDCA7, and HELLS, it highlights the probable existence of an independent pathway of DNMT3b-mediated demethylation. 

Further studies are needed to understand the precise role of each protein in the normal demethylation process, and the molecular mechanisms and differences between this hypothetical cooperative pathway and DNMT3B-mediated ZBTB24/CDCA7/HELLS-independent demethylation.

##### Common Variable Immunodeficiency

CVID is the most frequent PID. It is characterized by a reduction in B cell function, including impaired antibody generation (hypogammaglobulinemia), and reduced numbers of isotype-switched memory B cells. Usually developing during adulthood, the genetic basis of the disease is largely unknown, and are 5–25% cases with familial heritability [167]. This scenario, in keeping with the aforementioned regulation of B cell differentiation by epigenetic mechanisms, claimed scientific interest towards the role of DNA methylation in this context. When possible, in these cases, the study of discordant MZ twins allows the genetic and epigenetic determinants of diseases to be distinguished. 

To this end, genome-wide DNA methylation profiling of B cells from these patients was conducted in two different studies, which revealed a significant impairment in the demethylation of B cell genes in CVID twins. This blockage is matched by inhibition of the transcriptional activation of genes in the same regions. Analysis of naïve and memory B cell stages revealed that the greatest differences occurred in memory cells, which displayed higher methylation levels, decreased expression, and a loss of activating histone modifications like H3K4me3 in those regions in CVID. Those results reflect an epigenetic impairment of the naïve-to-memory transition in this context [168]. 

A more recent study noted differences among various CVID subphenotypes, suggesting that impairment occurs at different stages of B-cell fate determination. The degree of impairment in the demethylation of B cell genes is associated with the reduction of proliferation of the memory compartment. Hypermethylation of the *AICDA* locus in CVID patients is strongly correlated with the impairment of SHM, suggesting an epigenetic control of AID expression [169]. Further research is needed to establish whether defects in demethylation are a causal pathogenic mechanism or a consequence of impaired B cell function in this disease. 

#### 3.2.2. Innate Immune Deficiencies

The innate immune system relies on receptors called PRRs (pattern recognition receptors) which can recognize general patterns of microbial infection (PAMPs, pathogen-associated molecular patterns) or damage (DAMPs, danger-associated molecular patterns), such as lipopolysaccharide or extracellular ATP, respectively. 

An innate immune deficiency is a disease directly affecting the innate immune response. For instance, there are deficiencies in PRR pathway genes, such as those of the TLR4 pathway (IRAK4 deficiency [121,122] and MyD88 deficiency [170]) and the TLR3 pathway (toll-like receptor 3 (TLR3) deficiency [171] and TRAF3 deficiency).

Another family of innate immune deficiencies is made up of those with Mendelian susceptibility to mycobacterial diseases. In these diseases, the IL12/IFNγ axis, which is crucial to macrophage/lymphocyte crosstalk, is affected, producing recurrent infections of mycobacteria. Several mutations have been described in patients in different genes: *IRF8*, *STAT1*, *IFNGR1*, *IFNGR2*, *STAT1*, *IL12B*, *IL12RB1*, *TYK2*, *CYBB*, and *IKBKG* [172].

Since immunodeficiencies of the innate immune system are generally monogenic diseases, with known genetic bases and a specific signaling pathway affected, there have been no studies of the methylation changes that could occur. 

However, given that signaling pathways affected in these diseases, such as that of TLR4 (LPS), have been described in some cases to cause modifications in DNA methylation, we cannot rule out the possibility that DNA methylation has a mechanistic role in the pathogenesis of the disease, conceivably modulating the course of the pathology. 

## 4. Conclusions

DNA methylation is crucial to the control of the immune system and its dysregulation is related to the pathogenesis of several immune system disorders. In this review, we have summarized the involvement of DNA methylation in regulating the differentiation, maturation, and function of the main cell types of the immune system. Immune system disorders can be classified as underactivation (immunodeficiencies) or overactivation (autoimmune/autoinflammatory diseases), and predominantly affect the innate immune or the adaptive immune responses. In fact, the reality is more complex, with diseases showing both immunodeficiency and autoimmunity, and a spectrum between innate immune and adaptive immune response defects. We have selected several diseases that typify the four aspects of this diversity: immunodeficiencies of the innate immune response, immunodeficiencies of the adaptive immune response, autoimmune and autoinflammatory diseases, and we have described the dysregulation of DNA methylation in these diseases. With these examples, we hope to provide a perspective on the wide range of DNA methylation defects, and the mechanisms potentially involved in generating them. These defects affect not only the underlying mechanisms of the disease, but also the possibility of using them as clinical biomarkers to monitor such changes.

## Figures and Tables

**Table 1:** Selected examples of DNA methylation implications in immune disease.

Immune Activity	Response Affected	Disease	Monogenic/Complex	Etiology	Cell types with Known DNA Methylation Defects
**Overactivation**	Adaptive	RA	Polygenic (20–50% heritability). HLA association: DRB1*01 and *04. Non-HLA SNP association: PTPN22, IL23R, TRAF1, CTLA4	Mostly unknown. Disease risk factors: age, cigarette smoke	Whole blood [100], PBLs [98], T cells [101,102], Monocytes [102,103]; B-cells [102], Fibroblast-like synoviocytes [104,105,106,107]
		SLE	Polygenic (4–20% heritability). HLA association: DRB1*1501 and DRB1*0301. Non-HLA SNP association: IRFs, STAT4, IFIH1, OPN	Mostly unknown. Disease risk factors: ultraviolet light, cigarette smoke	Whole Blood [108,109,110], T cells [111,112], B cells [113,114], Monocytes^118^
	Innate	CAPS	Monogenic	*NRLP3* GoF mutations	Monocytes [115]
		FMF	Monogenic	*MEFV* GoF mutations	PBMCs [116,117]
**Defect**	Adaptive	ICF	Monogenic	*DNMT3B* LoF mutations	Fibroblasts [118], Lymphoblastoid Cell Line [118,119,120]
		CVID	Polygenic (5–25% heritability)	Mostly unknown. Patients may present SNPs/mutations in *CTLA4, LRBA, TNFRSF13B, MSH5*	B Cells [121,122]
